# Medicaid Unwinding and Changes in Buprenorphine Dispensing

**DOI:** 10.1001/jamanetworkopen.2025.8469

**Published:** 2025-05-02

**Authors:** Joanne Constantin, Genevieve M. Kenney, Kosali Simon, Kao-Ping Chua

**Affiliations:** 1 Susan B. Meister Child Health Evaluation and Research Center, Department of Pediatrics, University of Michigan Medical School, Ann Arbor; 2 Health Policy Center, Urban Institute, Washington, DC; 3 Paul H. O’Neill School of Public and Environmental Affairs, Indiana University, Bloomington; 4 Department of Health Management and Policy, University of Michigan School of Public Health, Ann Arbor

## Abstract

**Importance:**

After the Medicaid continuous enrollment provision ended on March 31, 2023, millions of Medicaid patients were disenrolled, a process called “Medicaid unwinding.” Whether this process was associated with changes in dispensing of buprenorphine, a medication for opioid use disorder preventing opioid overdose deaths, is unknown.

**Objective:**

To evaluate changes in buprenorphine dispensing during Medicaid unwinding.

**Design, Setting, and Participants:**

This cross-sectional study used difference-in-differences analysis to assess data from the IQVIA Longitudinal Prescription Database, capturing 92% of US retail prescriptions. Treatment and comparison states were those in the top vs bottom quartile of the percentage change in adult Medicaid enrollment between the month before the state resumed Medicaid eligibility determinations and December 31, 2023. Analyses included Medicaid-insured adults with active buprenorphine prescriptions in quarter 1 from 2017 to 2023.

**Exposure:**

State-level change in adult Medicaid enrollment. The preexposure period was from July 1, 2017, to December 31, 2022, and the postexposure period was from July 1 to December 31, 2023 (quarters 3 and 4).

**Main Outcomes and Measures:**

Four outcomes assessed buprenorphine dispensing in quarters 3 and 4 from 2017 to 2023: the number of days with active buprenorphine prescriptions, no active prescriptions, 1 or more active prescriptions paid with private insurance, and 1 or more active cash-pay prescriptions. Linear and logistic regression models compared changes in outcomes over time among treatment and comparison states.

**Results:**

Analyses included 754 675 person-years from 569 069 patients (mean [SD] age, 39.2 [9.6] years; 386 719 men [51.2%]). Among adults in treatment states, the number of days with active buprenorphine prescriptions in quarters 3 and 4 decreased by 3.9 days (95% CI, −6.7 to −1.1 days) more compared with adults in comparison states. Adults in treatment states also had an increase of 1.8 percentage points (95% CI, 0.6-3.0 percentage points) in the probability of having no days with active prescriptions, an increase of 1.9 percentage points (95% CI, 0.4-3.4 percentage points) in the probability of having 1 or more active prescriptions paid with private insurance, and an increase of 0.9 percentage points (95% CI, 0.1-1.7 percentage points) in the probability of having 1 or more active cash-pay prescriptions.

**Conclusions and Relevance:**

In this cross-sectional study using difference-in-differences analysis, Medicaid patients in states with the highest vs lowest magnitude of Medicaid disenrollment through December 2023 were more likely to decrease or discontinue buprenorphine use and more likely to transition to private insurance or cash to pay for prescriptions. Findings suggest that Medicaid unwinding was associated with disruptions in buprenorphine therapy, raising concerns about the potential for increased opioid-related morbidity and mortality among patients in this population.

## Key Points

**Question:**

Among Medicaid-insured adults with buprenorphine use, were changes in buprenorphine dispensing greater among those residing in states with the highest vs lowest decreases in Medicaid enrollment after “Medicaid unwinding” began in April 2023?

**Findings:**

This cross-sectional study of 2017-2023 data from a national prescription dispensing database included 754 675 person-years from 569 069 patients. The study found that patients in states with the highest decreases in Medicaid enrollment were more likely to decrease buprenorphine use, discontinue buprenorphine therapy, and use private insurance or cash to pay for buprenorphine prescriptions.

**Meaning:**

This study suggests that Medicaid unwinding was associated with disruptions in buprenorphine therapy, raising concerns about the potential for increased opioid-related morbidity and mortality.

## Teaser

This cross-sectional study used data from a national prescription dispensing database to evaluate changes in buprenorphine dispensing after the Medicaid continuous enrollment provision ended on March 31, 2023.

## Introduction

The Families First Coronavirus Response Act, enacted in March 2020, provided increased federal funding to states maintaining continuous Medicaid enrollment during the COVID-19 public health emergency.^1,2,3,4,5,6^ Medicaid enrollment thus increased by 21.2 million between February 2020 and December 2022.^1,7^ The Consolidated Appropriations Act of 2022 ended the continuous enrollment provision effective March 31, 2023, allowing states to resume Medicaid eligibility redeterminations in a process that has been called “Medicaid unwinding.”^8^

As of July 23, 2024, a total of 24 million individuals in the US have been disenrolled from Medicaid since the unwinding process began.^9^ Approximately 70% of disenrollments were due to procedural issues, such as incomplete paperwork or failure to reach an enrollee, rather than a determination of ineligibility.^9^ Moreover, the magnitude of disenrollment has varied widely by state, partly owing to differences in approaches to unwinding and Medicaid eligibility criteria among states.^10,11,12^

Large-scale Medicaid disenrollment could have deleterious consequences for the US opioid epidemic if it impedes access to effective treatments for opioid use disorder, such as buprenorphine prescription.^13^ To date, however, we are unaware of any national studies evaluating this possibility. In this study, we used a national prescription dispensing database to evaluate whether adult Medicaid patients with buprenorphine use were more likely to decrease or discontinue buprenorphine use if they lived in states with the highest vs lowest changes in Medicaid enrollment through the end of 2023. We also evaluated whether they were more likely to transition to private insurance or cash to pay for buprenorphine prescriptions, potentially increasing financial burden.

## Methods

### Data Source

Data on buprenorphine dispensing for this study were obtained from the IQVIA Longitudinal Prescription Database, which captures 92% of prescriptions dispensed from US retail pharmacies. The database includes unique patient identifiers, allowing for longitudinal tracking of patient prescription activity. The database does not report race or ethnicity.^14^ The method of payment for prescriptions is classified as Medicaid, Medicare, cash, and commercial, the latter of which includes prescriptions paid using coupons, vouchers, or discount cards. Data on monthly adult Medicaid enrollment were derived from the Georgetown University Center for Children and Families (eMethods 1 in Supplement 1).^15,16^ As data were deidentified, the institutional review board of the University of Michigan Medical School exempted this study from human participants review, and informed consent was not required. This manuscript follows the Strengthening the Reporting of Observational Studies in Epidemiology (STROBE) reporting guideline for cross-sectional studies.^17^

### Study Design

The study used a difference-in-differences design leveraging state-level variation in changes in adult Medicaid enrollment during unwinding. Alternatives to this dose-response approach were considered, including leveraging variation in the month when states began Medicaid unwinding, but this strategy was infeasible because almost all states began unwinding during the 4-month period between April and July 2023. The preintervention period was from July 1, 2017, to December 31, 2022, and the postintervention period was from July 1 to December 31, 2023. The study outcomes assessed buprenorphine dispensing in quarters 3 and 4, with the single postintervention data point (quarters 3 and 4 of 2023) derived from a period that occurred after unwinding began in quarter 2 of 2023. The preintervention years (2017-2019) and during the pandemic (2020-2022) were equal in length.

States were eligible if they began Medicaid unwinding from April to July 2023 and did not enact other policies expanding adult Medicaid enrollment in 2023. Oregon was ineligible because it did not begin unwinding until October 2023. Georgia, South Dakota, and North Carolina were ineligible because they enacted Medicaid expansions in 2023.^18^ For the remaining 46 states and the District of Columbia, the percentage change in adult Medicaid enrollment between the month before the state resumed Medicaid eligibility determinations (baseline month) and December 2023 was calculated. Treatment and comparison states were those in the top vs bottom quartiles of this percentage change. eMethods 1 in Supplement 1 includes additional details on assignment of states to treatment and comparison groups.

### Sample

For each year from 2017 to 2023, the sample included adults aged 18 to 64 years with at least 1 active Medicaid-paid buprenorphine prescription in quarter 1. A buprenorphine prescription was considered active from the dispensing date through the end date, defined as dispensing date plus the number of days supplied minus 1. Patients could have active buprenorphine prescriptions in quarter 1 even if they did not have any buprenorphine dispensing during quarter 1 (eg, if the active period of a prescription dispensed in the preceding December extended into quarter 1). Patients could contribute data to multiple years. Adults aged 65 years or older with Medicaid-paid buprenorphine prescriptions were excluded because they could be dually eligible for Medicare and thus could still have alternative prescription drug coverage even if disenrolled from Medicaid.

### Study Variables

The exposure was an indicator for residence in a state in the top vs bottom quartile of the percentage change in adult Medicaid enrollment. There were 4 person-year–level outcomes measuring buprenorphine dispensing in quarters 3 and 4: number of days with active buprenorphine prescriptions (regardless of payment method), indicator for having no active buprenorphine prescription, indicator for having 1 or more active buprenorphine prescriptions paid with private insurance, and indicator for having 1 or more active buprenorphine prescriptions paid with cash. The first 2 outcomes assessed the intensity and occurrence of buprenorphine use, while the third and fourth outcomes assessed potential transitions from Medicaid to private insurance and no insurance, respectively.

### Statistical Analysis

Linear or logistic regression difference-in-differences models were used to calculate the difference in the change in outcomes between 2017 to 2022 and 2023 among treatment vs comparison states. Models included the interaction between indicators for treatment group and postintervention period (coefficient of interest), state fixed effects, year fixed effects, and patient age and sex. For logistic regression models, average marginal effects were calculated to allow interpretation of difference-in-differences estimates as absolute changes in probability. All models used robust SEs clustered at the state level. eMethods 2 in Supplement 1 includes additional details on model specification.

To assess the parallel trends assumption (ie, treatment and comparison groups’ outcomes moving in tandem before 2023), event study models were fitted. These models included fixed effects for each year other than 2022, the interaction between these fixed effects and the indicator for treatment group, state fixed effects, and patient age and sex. The parallel trends assumption would be supported if none of the pre-2022 interaction terms were different from zero. See eMethods 3 in Supplement 1 for additional details.

Statistical analyses were conducted using Stata, version 18.1/MP (StataCorp LLC).^19^ Two-sided hypothesis tests were conducted with α = .05.

#### Subgroup Analyses

First, analyses were repeated among person-years from patients with 60 or more vs less than 60 days of active buprenorphine prescriptions during quarter 1. The 60-day cutoff was used because some definitions of buprenorphine retention in therapy allow for up to a 30-day dispensing gap.^20,21^ Second, analyses were repeated among person-years from patients with only branded prescriptions in quarter 1 vs all other patients (ie, those only having generic buprenorphine prescriptions or a mixture of branded and generic prescriptions). This second analysis included data from 2020 to 2023 because almost all buprenorphine dispensing was for branded products until 2019, when a generic formulation of buprenorphine-naloxone film entered the market.^22,23^

#### Sensitivity Analyses

First, analyses were repeated when excluding Texas and California, 2 populous states in the treatment and comparison groups, respectively. Second, analyses were repeated when limited to data from 2020 to 2023, ensuring the preintervention period occurred during the COVID-19 pandemic. Third, analyses were repeated when measuring buprenorphine dispensing outcomes only in quarter 4. Fourth, analyses were repeated when counting buprenorphine prescriptions dispensed only in quarters 3 and 4, thus excluding prescriptions dispensed before whose active period extended into quarters 3 and 4 (see eMethods 4 in Supplement 1 for details).

## Results

### Sample Characteristics

Table 1 shows sample characteristics overall and by group. Overall, there were 754 675 person-years of data from 569 069 patients (mean [SD] age, 39.2 [9.6] years; 386 719 men [51.2%] and 367 956 women [48.8%]). Among the 754 675 person-years of data, 233 704 person-years (31.0%) were from patients in treatment states, and 435 385 person-years (57.7%) were from patients who had 60 days or more with active buprenorphine prescriptions in quarter 1. Among 513 494 person-years from 2020 to 2023, 318 813 (62.1%) were from patients who only had active branded buprenorphine prescriptions in quarter 1. eTable 1 in Supplement 1 shows sample sizes in each year both overall and by group.

**Table 1.** Sample Characteristics

Characteristic	No. (%) of person-years of data^a^		
	Total sample (N = 754 675 [100%])	Treatment group (n = 233 704 [31.0%])	Comparison group (n = 520 971 [69.0%])
Age, mean (SD), y	39.2 (9.6)	38.3 (9.1)	39.6 (9.8)
Age group, y			
18-25	30 971 (4.1)	10 793 (4.6)	20 178 (3.9)
26-34	245 135 (32.5)	79 438 (34.0)	165 697 (31.8)
35-44	279 988 (37.1)	90 968 (38.9)	189 020 (36.3)
45-54	129 091 (17.1)	36 331 (15.5)	92 760 (17.8)
55-64	69 490 (9.2)	16 174 (6.9)	53 316 (10.2)
Sex			
Male	386 719 (51.2)	107 955 (46.2)	278 764 (53.5)
Female	367 956 (48.8)	125 749 (53.8)	242 207 (46.5)

^a^ The numbers refer to the number of person-years of data, not the number of patients, as a given patient could account for multiple person-years of data (eg, if they had active Medicaid-paid buprenorphine prescriptions in both quarter 1 of 2017 and quarter 1 of 2018). Treatment states include Arkansas, Colorado, Idaho, Kansas, Montana, New Hampshire, North Dakota, Oklahoma, Texas, Utah, West Virginia, and Wyoming. Comparison states include California, Connecticut, Delaware, Hawaii, Illinois, Maine, Massachusetts, Minnesota, Nebraska, Nevada, Virginia, and Wisconsin.

### Main Analysis

The Figure shows trends in unadjusted outcomes by year in treatment and comparison states. Table 2 displays unadjusted outcomes in the treatment and comparison groups from 2017 to 2022 vs 2023, as well as the difference-in-differences estimates. Among person-years from patients in treatment states, the mean (SD) number of days with active buprenorphine prescriptions in quarters 3 and 4 decreased from 104.4 (76.7) to 99.9 (76.4), compared with a decrease from a mean of 103.1 (76.2) to 102.6 (75.1) among person-years from patients in comparison states (difference-in-differences estimate, −3.9 days [95% CI, −6.7 to −1.1 days]; *P* = .009). Among person-years from patients in treatment states, the proportion with no active buprenorphine prescriptions in quarters 3 and 4 increased from 23.4% to 24.6% compared with a decrease from 24.1% to 23.6% in comparison states (difference-in-differences estimate, 1.8 percentage points [95% CI, 0.6-3.0 percentage points]; *P* = .004). Among person-years from patients in treatment states, the proportion with 1 or more active buprenorphine prescriptions paid with private insurance in quarters 3 and 4 increased from 11.0% to 15.9% compared with an increase from 8.7% to 9.6% in comparison states (difference-in-differences estimate, 1.9 percentage points [95% CI, 0.4-3.4 percentage points]; *P* = .01). Among person-years from patients in treatment states, the proportion with 1 or more active cash-pay buprenorphine prescriptions in quarters 3 and 4 increased from 5.4% to 6.2% compared with a decrease from 2.8% to 2.4% in comparison states (difference-in-differences estimate, 0.9 percentage points [95% CI, 0.1-1.7 percentage points]; *P* = .03). Event study analyses supported the parallel trends assumption for all outcomes except for a minor violation in 2018 and 2021 for the cash-pay buprenorphine dispensing outcome (eFigure in Supplement 1).

**Figure.**
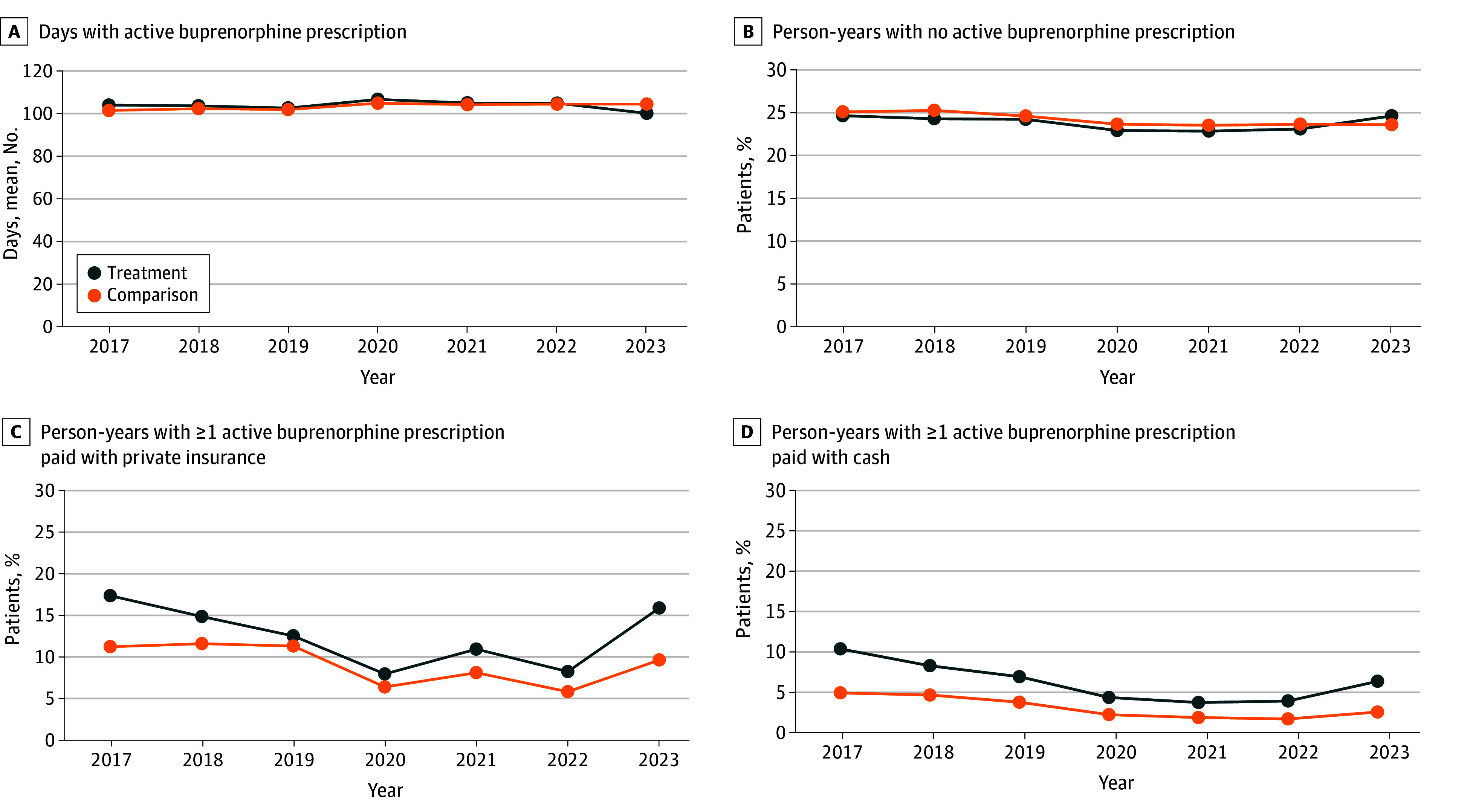
Trends in Unadjusted Outcomes in Treatment and Comparison States Between 2017 and 2023 A, Mean number of days with active buprenorphine prescriptions in quarters 3 and 4. B, Proportion of person-years with no active buprenorphine prescriptions in quarters 3 and 4. C, Proportion of person-years with 1 or more active buprenorphine prescriptions paid with private insurance in quarters 3 and 4. D, Proportion of person-years with 1 or more active buprenorphine prescriptions paid with cash in quarters 3 and 4. Treatment states include Arkansas, Colorado, Idaho, Kansas, Montana, North Dakota, New Hampshire, Oklahoma, Texas, Utah, West Virginia, and Wyoming. Comparison states include California, Connecticut, Delaware, Hawaii, Illinois, Maine, Massachusetts, Minnesota, Nebraska, Nevada, Virginia, and Wisconsin.

**Table 2.** Difference-in-Differences Estimates of the Association Between the Magnitude of Disenrollment After Medicaid Unwinding Began and Buprenorphine Dispensing^a^

Outcome of active buprenorphine prescription in Q3 and Q4	Unadjusted outcome, %				Adjusted difference-in-differences estimate (95% CI)
	Treatment states		Comparison states		
	Preintervention	Postintervention	Preintervention	Postintervention	
No. of days, mean (SD)	104.4 (76.7)	99.9 (76.4)	103.1 (76.2)	102.6 (75.1)	−3.9 (−6.7 to −1.1)^b^
No active prescription	23.4	24.6	24.1	23.6	1.8 (0.6 to 3.0)^b^
≥1 Active prescription paid by private insurance	11.0	15.9	8.7	9.6	1.9 (0.4 to 3.4)^c^
≥1 Active prescription paid by cash	5.4	6.2	2.8	2.4	0.9 (0.1 to 1.7)^c^

Abbreviation: Q, quarter.

^a^ Treatment states include Arkansas, Colorado, Idaho, Kansas, Montana, New Hampshire, North Dakota, Oklahoma, Texas, Utah, West Virginia, and Wyoming. Comparison states include California, Connecticut, Delaware, Hawaii, Illinois, Maine, Massachusetts, Minnesota, Nebraska, Nevada, Virginia, and Wisconsin.

^b^ *P* < .01.

^c^ *P* < .05.

### Subgroup Analyses

Table 3 displays the difference-in-differences estimates from subgroup analyses. Among person-years from patients with less than 60 days with active buprenorphine prescriptions in quarter 1, the differential change between the treatment and comparison groups in the number of days with active buprenorphine prescriptions in quarters 3 and 4 was −5.8 (95% CI, −9.6 to −2.1; *P* = .004). The differential change in the probability of having no active prescriptions was 3.2 percentage points (95% CI, 1.7-4.8 percentage points; *P* < .001).

**Table 3.** Difference-in-Differences Estimates of the Association Between the Magnitude of Disenrollment After Medicaid Unwinding Began and Buprenorphine Dispensing in Subgroup Analyses^a^

Outcome of active buprenorphine prescription in Q3 and Q4	Difference-in-differences estimate as absolute change in probability (95% CI)				
	<60 d (n = 319 290)^b^	≥60 d (n = 435 385)^b^	Only active branded prescriptions (n = 318 813)^b^	Only generic or mixture of generic and branded prescriptions (n = 194 681)^b^
No. of days, mean (95% CI)	−5.8 (−9.6 to −2.1)^c^	−1.0 (−4.3 to 2.4)	−5.0 (−9.7 to −0.4)^d^	−1.1 (−5.0 to 2.7)
No active prescription	3.2 (1.7 to 4.8)^c^	0.0 (−1.0 to 1.1)	2.2 (0.01 to 4.4)^d^	0.6 (−1.3 to 2.5)
≥1 Active prescription paid by private insurance	1.2 (−0.6 to 2.9)	2.3 (0.2 to 4.4)^d^	3.2 (1.6 to 4.9)^c^	0.8 (−2.8 to 4.4)
≥1 Active prescription paid by cash	0.5 (−0.2 to 1.2)	1.2 (0.3 to 2.1)^c^	0.4 (−0.3 to 1.1)	0.8 (−0.3 to 1.8)

Abbreviation: Q, quarter.

^a^ Treatment states include Arkansas, Colorado, Idaho, Kansas, Montana, New Hampshire, North Dakota, Oklahoma, Texas, Utah, West Virginia, and Wyoming. Comparison states include California, Connecticut, Delaware, Hawaii, Illinois, Maine, Massachusetts, Minnesota, Nebraska, Nevada, Virginia, and Wisconsin.

^b^ Defined as having active buprenorphine prescriptions in Q1.

^c^ *P* < .01.

^d^ *P* < .05.

Among person-years from patients with 60 days or more with active buprenorphine prescriptions in quarter 1, the differential change between the treatment and comparison groups in the probability of having 1 or more active prescriptions paid with private insurance was 2.3 percentage points (95% CI, 0.2-4.4 percentage points; *P* = .03) (Table 3). The differential change in the probability of having 1 or more active prescriptions paid with cash was 1.2 percentage points (95% CI, 0.3-2.1 percentage points; *P* = .008). No other difference-in-differences estimates were significant.

Among person-years from patients with only branded prescriptions in quarter 1, the differential change between the treatment and comparison groups in the number of days with active prescriptions in quarters 3 and 4 was −5.0 days (95% CI, −9.7 to −0.4 days; *P* = .04) (Table 3). The differential change for the remaining 3 outcomes was positive (no active prescriptions: 2.2 percentage points [95% CI, 0.01-4.4 percentage points; *P* = .049]; ≥1 active prescription paid with private insurance: 3.2 percentage points [95% CI, 1.6-4.9 percentage points; *P* < .001]; ≥1 active prescription paid with cash: 0.4 percentage points [95% CI, −0.3 to 1.1 percentage points; *P *= .22]), although the finding was nonsignificant for the cash-pay outcome. In contrast, among person-years from other patients, there was no differential change in any outcome.

### Sensitivity Analysis

Conclusions were qualitatively unchanged in sensitivity analyses, except that the differential increase in the probability of having 1 or more prescriptions paid with cash became nonsignificant when excluding Texas and California (0.8 percentage points [95% CI, −0.2 to 1.7 percentage points]) and when limiting to 2020-2023 data (0.4 percentage points [95% CI, −0.1 to 0.9 percentage points]) (eTable 2 in Supplement 1). Moreover, the difference-in-difference estimate for the probability of having 1 or more cash-pay buprenorphine prescriptions dispensed in quarters 3 and 4 was positive but nonsignificant (0.8 percentage points [95% CI, −0.01 to 1.6 percentage points]).

## Discussion

In this national study of adult Medicaid patients with buprenorphine dispensing, those in states with the greatest enrollment changes through December 2023 experienced a larger decrease in the intensity and occurrence of buprenorphine dispensing in quarters 3 and 4 and a greater increase in the use of private insurance or cash to pay for buprenorphine prescriptions compared with adults in states with the smallest enrollment changes. Findings suggest Medicaid unwinding was associated with disruptions in buprenorphine therapy, raising concerns about the potential for increased opioid-related morbidity and mortality in this population.

A large body of literature has shown that Medicaid coverage loss is associated with greater cost-related barriers to accessing care and worsened medication adherence.^24,25,26,27,28,29^ To our knowledge, this study is one of the first to suggest that similar changes occurred in the context of Medicaid unwinding. Although the reported associations were small in magnitude, they reflect all patients in treatment and comparison states, not the subset of patients who were disenrolled. If the database included information on disenrollment, allowing the analysis to be limited to this subset, estimates of changes in buprenorphine dispensing likely would have been considerably larger.

Medicaid patients in treatment states experienced a greater decrease in the number of days with active buprenorphine prescriptions and a greater increase in the probability of having no active prescriptions relative to those in comparison states. This finding suggests Medicaid unwinding might be contributing to the ongoing underuse of buprenorphine and the low rates of buprenorphine retention among patients with opioid use disorder.^30,31,32^ Moreover, given the association between buprenorphine retention and decreased risk of opioid overdose mortality,^33^ Medicaid unwinding may also be exacerbating the US epidemic of opioid overdose deaths.

The greater use of private insurance and cash to pay for buprenorphine prescriptions among adults in treatment vs comparison states is not unexpected, as studies suggest that many of the patients disenrolled from Medicaid during the unwinding transitioned to private insurance and cash.^34,35,36^ However, these transitions raise concerns about increased financial barriers to accessing buprenorphine. For example, a survey of adult Medicaid enrollees with low income in 4 Southern states revealed that individuals who became uninsured or transitioned to alternative coverage sources were more likely to report delays in medication receipt due to cost compared with those who remained enrolled in Medicaid.^35^ The increased transition to cash payment is particularly concerning, as patients using cash may be less likely to continue therapy given greater exposure to cost sharing.

Among patients with fewer than 60 days of buprenorphine use in quarter 1, those residing in treatment vs comparison states were more likely to have decreases in the intensity and occurrence of buprenorphine use in quarters 3 and 4 but were not more likely to have prescriptions paid by private insurance or cash. In contrast, among patients with 60 or more days of buprenorphine use in quarter 1, those residing in treatment vs comparison states were not more likely to have changes in the intensity or occurrence of buprenorphine use, but they were more likely to have prescriptions paid with private insurance or cash. These findings suggest that the demand for buprenorphine is less sensitive to cost sharing among patients with greater baseline use and more sensitive to cost sharing among those with less baseline use, perhaps because the latter group of patients is only beginning buprenorphine therapy and has not yet experienced its benefits. This possibility is supported by a prior study suggesting that the association between cost sharing and the probability of buprenorphine prescription nondispensing is slightly larger among patients initiating buprenorphine therapy.^37^

Among patients using only branded formulations of buprenorphine in quarter 1, those residing in treatment vs comparison states were more likely to experience decreases in the intensity and occurrence of buprenorphine use in quarters 3 and 4 and more likely to use private insurance to pay for buprenorphine prescriptions. In contrast, among patients using only generic formulations or a mixture of generic and branded formulations, those residing in treatment vs comparison states were not more likely to use cash or private insurance to pay for prescriptions or to experience changes in the intensity and occurrence of buprenorphine use. For the former, it is possible that the high price of branded buprenorphine impeded dispensing and deterred patients from paying out of pocket with cash if they lost Medicaid coverage. Future studies might evaluate whether similar findings occurred for patients using other branded medications for chronic conditions, suggesting that these patients may be an important target for efforts to connect patients who were disenrolled with alternative coverage sources.

As of the time of writing, Medicaid unwinding has been completed in most states.^9^ To mitigate any adverse changes to buprenorphine therapy associated with unwinding, states might consider several interventions. First, states and managed care plans could proactively engage patients with opioid use disorder who lost Medicaid coverage and connect them with Marketplace insurance plans or alternative safety net programs for which they may qualify. Second, states that have not expanded their Medicaid programs under the Affordable Care Act could consider doing so. Third, states could consider applying for waivers to require 12 months of continuous Medicaid coverage for adults; 5 states have already adopted this approach.^38^

### Limitations

This study has some limitations. First, the database did not allow for the identification of patients who were disenrolled from Medicaid during unwinding. Thus, we could only estimate the association between living in a state with high Medicaid enrollment loss and buprenorphine dispensing. This association is not the same as the association between disenrollment and buprenorphine dispensing, although it is likely similar in direction. Second, the study period did not capture the full period during which Medicaid unwinding occurred. Third, this study did not compare changes in opioid-related adverse events, such as opioid overdose, between treatment and comparison states. Fourth, estimates could be biased if other state policies differentially changed buprenorphine dispensing during the postintervention period (quarters 3 and 4 of 2023). However, we are unaware of any such policies. For example, while it is theoretically possible that states varied in their approach to implementing the elimination of the buprenorphine waiver requirement, both national and single-state studies suggest that the elimination of this requirement was not associated with changes in buprenorphine dispensing.^30,39^ Fifth, the degree to which adult Medicaid enrollment decreased during unwinding may have been associated with other factors potentially associated with buprenorphine use, such as the robustness of state’s private insurance market.

## Conclusions

In this national study, patients in states with greater enrollment loss after Medicaid unwinding began were more likely to decrease or discontinue buprenorphine use than those in states with smaller enrollment loss. Future research might identify whether specific unwinding-related policies protected patients from disruptions in buprenorphine therapy.

## Notes

Supplement 1. **eMethods 1.** Details on the Used Data Sources and on the Assignment of States Into Treatment and Comparison Groups **eMethods 2.** Difference-in-Differences Models **eMethods 3.** Event-Study Models **eMethods 4.** Sensitivity Analysis Using Alternative Outcomes **eFigure.** Event-Study Plots for the Association Between the Magnitude of Disenrollment After Medicaid Unwinding Began and Buprenorphine Dispensing **eTable 1.** Number of Individuals Observed in Q1 of Each Year (2017-2023), Overall and Stratified by Treatment Versus Comparison States **eTable 2.** Difference-in-Differences Estimates of the Association Between the Magnitude of Disenrollment After Medicaid Unwinding Began and Buprenorphine Dispensing – Results From Sensitivity Analyses

Supplement 2.
Data Sharing Statement
