# Supplementary material for: Medicaid Unwinding and Changes in Buprenorphine Dispensing
Source: JAMA Netw Open. 2025 May 2;8(5):e258469. doi: 10.1001/jamanetworkopen.2025.8469 (PMC12048846; doi:10.1001/jamanetworkopen.2025.8469)
Supplement: Supplement 1. — eMethods 1. Details on the Used Data Sources and on the Assignment of States Into Treatment and Comparison Groups eMethods 2. Difference-in-Differences Models eMethods 3. Event-Study Models eMethods 4. Sensitivity Analysis Using Alternative Outcomes eFigure. Event-Study Plots for the Association Between the Magnitude of Disenrollment After Medicaid Unwinding Began and Buprenorphine Dispensing eTable 1. Number of Individuals Observed in Q1 of Each Year (2017-2023), Overall and Stratified by Treatment Versus Comparison States eTable 2. Difference-in-Differences Estimates of the Association Between the Magnitude of Disenrollment After Medicaid Unwinding Began and Buprenorphine Dispensing – Results From Sensitivity Analyses [file jamanetwopen-e258469-s001.pdf]

Supplementary Online Content

Constantin J, Kenney GM, Simon K, Chua K-P. Medicaid unwinding and changes in buprenorphine dispensing. *JAMA Netw Open.* 2025;8(5):e258469. doi:10.1001/jamanetworkopen.2025.8469

eMethods 1. Details on the Used Data Sources and on the Assignment of States Into Treatment and Comparison Groups

eMethods 2. Difference-in-Differences Models

eMethods 3. Event-Study Models

eMethods 4. Sensitivity Analysis Using Alternative Outcomes

eFigure. Event-Study Plots for the Association Between the Magnitude of Disenrollment After Medicaid Unwinding Began and Buprenorphine Dispensing

eTable 1. Number of Individuals Observed in Q1 of Each Year (2017-2023), Overall and Stratified by Treatment Versus Comparison States

eTable 2. Difference-in-Differences Estimates of the Association Between the Magnitude of Disenrollment After Medicaid Unwinding Began and Buprenorphine Dispensing – Results From Sensitivity Analyses

This supplementary material has been provided by the authors to give readers additional information about their work.

© 2025 Constantin J et al. *JAMA Network Open.*

eMethods 1. Details on the Used Data Sources and on the Assignment of States Into Treatment and Comparison Groups

The IQVIA Longitudinal Prescription Database is a comprehensive national database that captures prescriptions dispensed from 92% of U.S. retail pharmacies and the majority of prescriptions dispensed from mail-order and long-term care pharmacies. The database includes prescriptions across all methods of payment, including cash. Data are at the prescription level and are not projected to national totals. The database includes information on all prescriptions dispensed from over 50,000 of the approximately 60,000 retail, mail-order, and long-term care pharmacies in the U.S. In most cases, prescription information derives directly from pharmacies, but in some cases, information derives from software vendors and "switches" (i.e., software companies that process claims submitted by pharmacies to insurers). Data are updated weekly. Upon receipt, IQVIA initiates an extensive quality control process that includes standardization of patient, product, prescriber, payer, and outlet identifiers through IQVIA's reference files; removing duplicate transactions; ensuring prescription dates are valid; and active monitoring of pharmacy status to ensure data reporting stability and demographic accuracy. The database only includes paid claims (i.e., those for dispensed prescriptions).

The database does not capture dispensing from pharmacies in the Veterans Administration or the Indian Health Service, or dispensing from pharmacies that are only open to patients of a particular health system (e.g., pharmacies for Kaiser Permanente). Moreover, the database does not capture medications administered or directly dispensed during a visit, such as injections of extended-release buprenorphine billed to a patient's medical rather than pharmacy benefit. The database does capture buprenorphine prescriptions written in Opioid Treatment Programs if the prescriptions were filled in a pharmacy captured by the IQVIA database.

The database includes an encrypted provider identifier that stays constant across years. The provider identifier comes from IQVIA's provider reference file, which contains information on 6.5 million health care professionals, of which approximately 2.3 million are authorized to write prescriptions. The provider reference file is derived from multiple inputs, including the American Medical Association, National Provider Identifier, Drug Enforcement Agency, and State License Number. As with all IQVIA reference files, quality control for the provider reference file relies on standard business rules along with intelligence from external data users.

The database also includes a non-identifiable patient identifier. IQVIA relies on a trusted third party to ensure confidentiality and accuracy of this identifier. The identifier is derived from first and last name, date of birth, sex, address, and other inputs. The identifier remains unchanged over time and across IQVIA databases and has no missingness.

This table displays the month in which unwinding began in each state. Data derived from the Georgetown University Center for Children and Families, State Medicaid/CHIP Enrollment Data.

**Unwinding start month**	**States**
April	Arkansas, Arizona, Idaho, New Hampshire, Oklahoma, and South Dakota.
May	Alaska, Connecticut, Florida, Iowa, Indiana, Kansas, Kentucky, Montana, Nebraska, New Mexico, Ohio, Pennsylvania, Utah, Washington, West Virginia, and Wyoming.

© 2025 Constantin J et al. *JAMA Network Open.*

June	Alabama, Colorado, District of Columbia, Delaware, Georgia, Hawaii, Louisiana, Massachusetts, Maryland, Maine, Missouri, North Carolina, North Dakota, Nevada, Rhode Island, South Carolina, Tennessee, Texas, Virginia, Vermont, and Wisconsin.
July	California, Illinois, Michigan, Minnesota, Mississippi, New Jersey, and New York.
October	Oregon

Below, we display the adult Medicaid enrollment totals in the month before each state began unwinding and December 2023, as well as the percentage change (excluding Georgia, North Carolina, South Dakota, and Oregon). Data was extracted in May 2024.

The Georgetown University Center for Children and Families used the most recent publicly available state Medicaid administrative data in 31 states and substituted Centers for Medicare & Medicaid Services (CMS) preliminary enrollment data in the remaining 20 states. Since these figures are directly reported by official government entities, we rely on them as the most authoritative sources available.

**State**	**Baseline month (2023)**	**Baseline adult Medicaid enrollment**	**Adult Medicaid enrollment in December 2023)**	**Change in adult Medicaid enrollment**	**% change in adult Medicaid enrollment**
Hawaii	May	296,670	302,367	-5,697	1.92
Maine	May	238,444	233,125	5,319	-2.23
Virginia	May	1,120,664	1,080,480	40,184	-3.59
California	June	10,422,297	9,956,265	466,032	-4.47
Minnesota	June	786,474	741,948	44,526	-5.66
Illinois	June	2,360,977	2,225,773	135,204	-5.73
Delaware	May	180,141	169,726	10,415	-5.78
Wisconsin	May	930,441	869,282	61,159	-6.57
Connecticut	April	636,353	594,374	41,979	-6.6
Nebraska	April	190,752	177,621	13,131	-6.88
Massachusetts	May	1,520,350	1,402,278	118,072	-7.77
Nevada	May	517,904	476,064	41,840	-8.08
Rhode Island	May	239,596	218,260	21,336	-8.9
South Dakota	March	58,392	53,182	5,210	-8.92
Alaska	April	161,409	146,473	14,936	-9.25
Missouri	May	734,130	665,423	68,707	-9.36
New York	June	4,589,445	4,128,279	461,166	-10.05

Louisiana	May	1,279,620	1,143,806	135,814	-10.61
District of Columbia	May	195,328	174,469	20,859	-10.68
Mississippi	June	363,850	323,877	39,973	-10.99
New Jersey	June	1,122,882	994,927	127,955	-11.4
Michigan	April	2,079,323	1,836,590	229,013	-11.67
Ohio	June	1,962,804	1,733,791	242,733	-11.67
Washington	April	1,255,057	1,104,402	150,655	-12
Tennessee	May	821,543	715,635	105,908	-12.89
Maryland	May	1,149,757	997,180	152,577	-13.27
Kentucky	April	991,984	854,584	137,400	-13.85
South Carolina	May	561,330	480,494	80,836	-14.4
New Mexico	April	573,926	490,568	83,358	-14.52
Indiana	April	1,089,206	930,591	158,615	-14.56
Vermont	May	129,123	109,998	19,125	-14.81
Iowa	April	422,190	359,567	62,623	-14.83
Arizona	March	1,458,747	1,237,561	221,186	-15.16
Alabama	May	600,369	506,103	94,266	-15.7
Pennsylvania	April	2,118,269	1,781,485	336,784	-15.9
Florida	April	2,998,662	2,462,819	535,843	-17.87
Kansas	April	118,586	96,560	22,026	-18.57
West Virginia	April	389,380	314,555	74,825	-19.22
Wyoming	April	31,281	25,140	6,141	-19.63
North Dakota	May	74,759	59,085	15,674	-20.97
Colorado	May	1,041,195	784,574	256,621	-24.65
Oklahoma	March	675,738	500,477	175,261	-25.94
Arkansas	March	670,246	485,090	185,156	-27.63
Idaho	March	206,648	149,102	57,546	-27.85
Montana	April	161,199	113,760	47,439	-29.43
Utah	April	268,721	188,774	79,947	-29.75
New Hampshire	March	146,544	94,961	51,583	-35.2
Texas	May	1,564,601	966,486	598,115	-38.23

Among the 47 eligible states (including District of Columbia), the top quartile of the percentage change in adult Medicaid disenrollment was 18.22163%, and the bottom quartile was 8.491854%. The treatment and comparison states included the 12 states in the top quartile and the 12 states in the bottom quartile, respectively. These states are listed below.

© 2025 Constantin J et al. *JAMA Network Open.*

State assignment	
**Treatment states** (top quartile=18.22163)	Arkansas, Colorado, Idaho, Kansas, Montana, North Dakota, New Hampshire, Oklahoma, Texas, Utah, West Virginia, and Wyoming.
**Comparison states** (bottom quartile=8.491854)	California, Connecticut, Delaware, Hawaii, Illinois, Maine, Massachusetts, Minnesota, Nebraska, Nevada, Virginia, and Wisconsin.
**Excluded states**	Georgia, North Carolina, Oregon, Alaska, Alabama, Arizona, District of Columbia, Florida, Iowa, Indiana, Kentucky, Louisiana, Maryland, Michigan, Missouri, Mississippi, New Jersey, New Mexico, New York, Ohio, Pennsylvania, Rhode Island, South Carolina, South Dakota, Tennessee, Vermont, and Washington.

© 2025 Constantin J et al. *JAMA Network Open.*

eMethods 2. Difference-in-Differences Models

The following regression model was estimated for the continuous outcome (number of days with active buprenorphine prescriptions in Q3-Q4):

$$Y_{ist} = \beta_0 + \beta_1 * Top\_Quartile_s * Post_t + \mathbf{X'_{ist}} + \boldsymbol{\tau_t} + \boldsymbol{\sigma_s} + \varepsilon_{ist} \quad (1)$$

Y is one of the buprenorphine dispensing outcomes for patient *i* in state *s* in Q3-Q4 of year *t*. *Top_Quartile* is a binary indicator that equals 1 for patients in treatment states and 0 for patients in comparison states. *Post* is a binary indicator that equals 1 for 2023 and 0 for 2017-2022. Thus, the interaction term only equals 1 if data derive from treatment states in 2023. The main difference-in-differences parameter is β_1, representing any differential change in outcomes between 2017-2022 to 2023 among patients in treatment versus comparison states. **X** is a vector of patient characteristics that includes age and sex. $\boldsymbol{\tau}$ is a vector of year fixed effects, $\boldsymbol{\sigma}$ is a vector of state fixed effects, and ε is an idiosyncratic error term.

Logistic regression models were used for the three binary outcomes. The specification was identical except that a logit link was used.

© 2025 Constantin J et al. *JAMA Network Open.*

eMethods 3. Event-Study Models

The following regression model was estimated for the continuous outcome:

$$Y_{ist} = \beta_0 + \sum (\beta_t * Top\_Quartile_s * Year_t) + \boldsymbol{X'_{ist}} + \boldsymbol{\tau_t} + \boldsymbol{\sigma_s} + \varepsilon_{ist} \quad (2)$$

Similar to model (1), *Y* is one of the buprenorphine dispensing outcomes for patient *i* in state *s* in Q3-Q4 of year *t*. *Top_Quartile* is a binary indicator that equals 1 for patients in treatment states and 0 for patients in comparison states. *Year* includes binary (0/1) indicators for each year from 2017-2021 and 2023; 2022 was the reference year. β_{2017}–β_{2021} represent differences in changes from 2017 through 2021 relative to 2022 between patients in treatment and comparison groups. If these coefficients are significant, this would suggest differential pre-intervention trends. **X** is a vector of patient characteristics that includes age and sex. $\boldsymbol{\tau}$ is a vector of year fixed effects, $\boldsymbol{\sigma}$ is a vector of state fixed effects, and ε is an idiosyncratic error term.

Logistic regression models were used for the three binary outcomes. The specification was identical except that a logit link was used.

© 2025 Constantin J et al. *JAMA Network Open.*

eMethods 4. Sensitivity Analysis Using Alternative Outcomes

Analyses were repeated when using an alternative set of outcomes, each of which assessed the days supplied across all prescriptions filled in Quarter 3-4. These outcomes included the number of days supplied, indicator for no days supplied, indicator for ≥1 days supplied from a prescription paid with private insurance, and indicator for ≥1 days supplied from a cash-pay prescription. A key difference between these outcomes and those from the main analysis are that the former only captured utilization from prescriptions dispensed in Quarter 3-4, whereas the main analysis also considered prescriptions dispensed in Quarter 2 if the active period extended into Quarter 3.

© 2025 Constantin J et al. *JAMA Network Open.*

eFigure. Event-Study Plots for the Association Between the Magnitude of Disenrollment After Medicaid Unwinding Began and Buprenorphine Dispensing

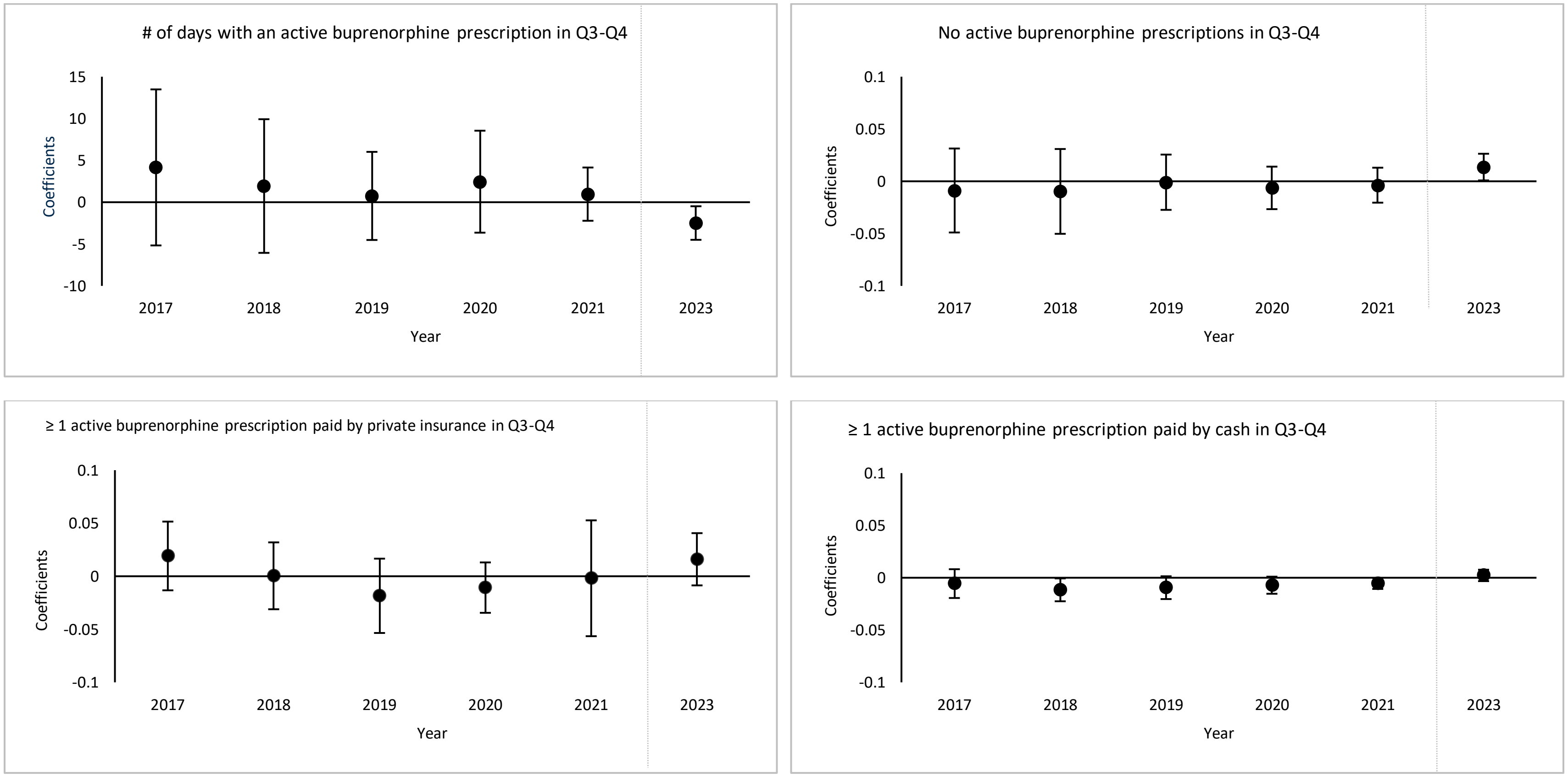

Event-study models included fixed effects for each year other than 2022, the interaction between these fixed effects and the indicator for treatment group, state fixed effects, and patient age and sex. For the number of days with active buprenorphine prescriptions, the coefficient is expressed in days. For all other outcomes, the coefficient is expressed as absolute percentage-point changes in probability. Treatment states include Arkansas, Colorado, Idaho, Kansas, Montana, North Dakota, New Hampshire, Oklahoma, Texas, Utah, West Virginia, and Wyoming. Comparison states include California, Connecticut, Delaware, Hawaii, Illinois, Maine, Massachusetts, Minnesota, Nebraska, Nevada, Virginia, and Wisconsin.

© 2025 Constantin J et al. *JAMA Network Open.*

eTable 1. Number of Individuals Observed in Q1 of Each Year (2017-2023), Overall and Stratified by Treatment Versus Comparison States

Year	Total sample	Treatment group	Year-over-year % change	Comparison group	Year-over-year % change
2017	66,480	16,790	N/A	49,690	N/A
2018	80,597	22,370	33.2%	58,227	17.2%
2019	94,104	27,497	22.9%	66,607	14.4%
2020	111,296	33,699	22.6%	77,597	16.5%
2021	121,948	39,083	16.0%%	82,865	6.8%
2022	133,021	44,909	14.9%	88,112	6.3%
2023	147,229	49,356	9.9%	97,873	11.1%

Treatment states include Arkansas, Colorado, Idaho, Kansas, Montana, North Dakota, New Hampshire, Oklahoma, Texas, Utah, West Virginia, and Wyoming.
Comparison states include California, Connecticut, Delaware, Hawaii, Illinois, Maine, Massachusetts, Minnesota, Nebraska, Nevada, Virginia, and Wisconsin.

© 2025 Constantin J et al. *JAMA Network Open.*

eTable 2. Difference-in-Differences Estimates of the Association Between the Magnitude of Disenrollment After Medicaid Unwinding Began and Buprenorphine Dispensing – Results From Sensitivity Analyses

Analysis	Number of days with an active buprenorphine prescription in Q3-Q4	No active buprenorphine prescription in Q3-Q4	At least one active buprenorphine prescription paid by private insurance in Q3-Q4	At least one active buprenorphine prescription paid by cash in Q3-Q4	N
Main analysis	-3.9^{***} [-6.7, -1.1]	1.8^{***} [0.6, 3.0]	1.9^{**} [0.4, 3.4]	0.9^{**} [0.1, 1.7]	754,675
Exclude California and Texas	-4.2^{**} [-7.4, -1.0]	2.0^{***} [0.7, 3.3]	1.9^{**} [0.1, 3.6]	0.8 [-0.2, 1.7]	640,562
Limit to 2020-2023 data	-3.5^{***} [-5.8, -1.1]	1.7^{***} [0.6, 2.7]	1.7^{**} [0.2, 3.1]	0.4 [-0.1, 0.9]	513,494
Limit post-period to Q4	-2.0^{***} [-3.4, -0.6]	2.3^{***} [0.9, 3.8]	2.0^{***} [0.8, 3.3]	0.6^{**} [0.00, 1.2]	754,675
Alternative outcomes	**Number of days supplied across buprenorphine prescriptions dispensed in Q3-Q4**	**No days supplied across buprenorphine prescriptions dispensed in Q3-Q4 (i.e., no fills)**	**At least one day supplied from a buprenorphine prescription paid by private insurance and dispensed in Q3-Q4**	**At least one day supplied from a buprenorphine prescription paid by cash and dispensed in Q3-Q4**	754,675
	-5.6^{***} [-8.6, -2.6]	1.9^{***} [0.6, 3.2]	1.8^{**} [0.4, 3.3]	0.8 [-0.01, 1.6]	

** $p < 0.05$, *** $p < 0.01$

The N in this table refers to the number of person-years of data, not the number of patients, as a given patient could account for multiple person-years of data (e.g., if they had active Medicaid-paid buprenorphine prescriptions in both Q1 of 2017 and Q1 of 2018).

Treatment states include Arkansas, Colorado, Idaho, Kansas, Montana, North Dakota, New Hampshire, Oklahoma, Texas, Utah, West Virginia, and Wyoming.

Comparison states include California, Connecticut, Delaware, Hawaii, Illinois, Maine, Massachusetts, Minnesota, Nebraska, Nevada, Virginia, and Wisconsin.

© 2025 Constantin J et al. *JAMA Network Open.*
