# Supplementary material for: Medicaid Unwinding and Changes in Buprenorphine Dispensing
Source: JAMA Netw Open. 2025 May 2;8(5):e258469. doi: 10.1001/jamanetworkopen.2025.8469 (PMC12048846; doi:10.1001/jamanetworkopen.2025.8469)
Supplement: Supplement 2. — Data Sharing Statement [file jamanetwopen-e258469-s002.pdf]

Data Sharing Statement

Constantin. Medicaid Unwinding and Changes in Buprenorphine Dispensing. *JAMA Netw Open*. Published May 02, 2025. doi:10.1001/jamanetworkopen.2025.8469

Data
Data available: No

Additional Information
Explanation for why data not available: IQVIA data are proprietary and cannot be shared.
